# Substance use disorders in adolescence - a review

**DOI:** 10.1192/j.eurpsy.2021.2183

**Published:** 2021-08-13

**Authors:** J. Mendes Coelho, C. Peixoto, M. Bicho, H. Fontes

**Affiliations:** Unidade De Agudos De Psiquiatria, Hospital do Divino Espírito Santo de Ponta Delgada, E.P.E., Ponta Delgada, Portugal

**Keywords:** Substance Use Disorder, Addiction, adolescence, Alcohol

## Abstract

**Introduction:**

Substance use disorders in adolescents are a growing problem worldwide. These disorders are often unrecognised, unvalued by families, society and clinicians and as a result underdiagnosed, with serious future consequences if improperly addressed.

**Objectives:**

Updated review of the recent literature on this topic.

**Methods:**

Unsystematic review of the most recent and relevant literature.

**Results:**

Review of neurobiology, risk factors, co-morbidity, differential diagnosis, diagnostic criteria, evaluation and treatment of substance use disorders in adolescence.

**Conclusions:**

Substance use disorder in adolescence includes a variety of behaviours related to the use of alcohol and/or drugs, for instance, inability to control substance use, impairment of function at school, home or work, interpersonal problems and hazardous use of substance. Further knowledge in identifying, early diagnosing and adequate intervention in adolescents’ substance use disorder may have paramount prognostic features.

**Disclosure:**

No significant relationships.

